# Effect of the Distillation Time on the Chemical Composition, Antioxidant Potential and Antimicrobial Activity of Essential Oils from Different *Cannabis sativa* L. Cultivars

**DOI:** 10.3390/molecules26164770

**Published:** 2021-08-06

**Authors:** Sara Palmieri, Francesca Maggio, Marika Pellegrini, Antonella Ricci, Annalisa Serio, Antonello Paparella, Claudio Lo Sterzo

**Affiliations:** Faculty of Bioscience and Technologies for Food, Agriculture and Environment, University of Teramo, Via R. Balzarini, 1, 64100 Teramo, Italy; spalmieri@unite.it (S.P.); fmaggio@unite.it (F.M.); mpellegrini@unite.it (M.P.); apaparella@unite.it (A.P.); closterzo@unite.it (C.L.S.)

**Keywords:** *Cannabis sativa* L., steam distillation time, GC-MS, FRAP-ABTS-DPPH assays, *Pseudomonas fluorescens* P34

## Abstract

Within the unavoidable variability of various origins in the characteristics of essential oils, the aim of this study was to evaluate the effect of the distillation time on the chemical composition and biological activity of *Cannabis sativa* essential oils (EOs). The dry inflorescences came from Carmagnola, Kompolti, Futura 75, Gran Sasso Kush and Carmagnola Lemon varieties from Abruzzo region (Central Italy), the last two being new cultivar here described for the first time. EOs were collected at 2 h and 4 h of distillation; GC/MS technique was applied to characterize their volatile fraction. The EOs were evaluated for total polyphenol content (TPC), antioxidant capacity (AOC) and antimicrobial activity against food-borne pathogens and spoilage bacteria. The time of distillation particularly influenced EOs chemical composition, extracting more or less terpenic components, but generally enriching with minor sesquiterpenes and cannabidiol. A logical response in ratio of time was observed for antioxidant potential, being the essential oils at 4 h of distillation more active than those distilled for 2 h, and particularly Futura 75. Conversely, except for Futura 75, the effect of time on the antimicrobial activity was variable and requires further investigations; nevertheless, the inhibitory activity of all EOs against *Pseudomonas fluorescens* P34 was an interesting result.

## 1. Introduction

Bioactive compounds are chemical components present in plants with interesting chemical and biological activities [1]. Already in ancient times, aromatic and medicinal plants were used to relieve symptoms and cure human diseases, thanks to their pharmacological properties due to the presence of these components [2].

In the last decades, several agricultural farms, research organizations and institutions have shown a new interest in the cultivation of *Cannabis sativa* L. with low content of Δ9-tetrahydrocannabinol THC (industrial hemp), because of its good application in food and pharmaceutical fields. In fact, *Cannabis sativa* L., a plant belonging to Cannabaceae family, has long been known to contain powerful antioxidant, antibacterial and antimicrobial agents as cannabinoids, terpenoids and polyphenols that act in synergy [3,4,5].

The hemp essential oil (EO) is secreted in glandular trichomes distributed on the epidermis of leaves and, to a major extent, on that of inflorescences [6]. The EO is characterized by a rich content of bioactive compounds, responsible for the potential biological activities. Among the bioactive compounds, terpenes are the most abundant in the volatile fraction, which is composed of monoterpenes (e.g., α-pinene, myrcene and terpinolene) and bitter-tasting sesqui-terpenes (e.g., caryophyllene, humulene and caryophyllene oxide) [7]. Among the latter, caryophyllene and its degradation product caryophyllene oxide showed important anticancer and analgesic properties [8]. The least volatile fraction of hemp essential oil is made up of cannabinoids, among which cannabidiol (CBD) is by far the predominant compound. CBD is formed from cannabidiolic acid (CBDA) through decarboxylation into the relative neutral form, which is the pharmacologically active one [9].

*Cannabis sativa* EOs are attracting the researchers’ attention, and studies are conducted to show its antioxidant and antimicrobial activity [6,10]. Regarding the antimicrobial activity, different studies are based on the evaluation of EOs obtained from different hemp cultivars. In detail, Zengin et al. [11] have demonstrated the antimicrobial effect of variety Futura 75 EO on bacterial strains isolated from clinical environment [11], while Nissen et al. [12] have tested the activity of EOs of three varieties of industrial hemp on Gram-positive and Gram-negative bacteria, concluding that hemp EO can significantly inhibit microbial growth [12]. Finally, Marini et al. [13] have tested the ability of *cv.* Futura 75 to reduce the virulent action of the food-borne pathogen *Listeria monocytogenes* [13].

Steam distillation is the most popular classical method to obtain essential oils and the time of distillation can play a pivotal role in determining the quality of hemp oils. To the best of our knowledge, in literature no reports are published concerning the possible enhancement of the biological activity due to the increase of distillation time.

To fill this gap, in the present work, EOs were collected at 2 h and 4 h of distillation from 5 different cultivars of hemp grown in Abruzzo region (Futura 75, Gran Sasso Kush, Carmagnola Lemon, Carmagnola and Kompolti); among them, Gran Sasso Kush and Carmagnola Lemon have been selected as new cultivar developed in this territory. The EOs were analyzed, evaluating how the time of distillation could influence the volatile fraction composition, the antioxidant capacity and the in vitro antimicrobial activity against food spoiling and pathogenic bacteria. Food-borne bacteria were selected, to evaluate the efficacy of the EOs in view of a potential application in the food industry as natural preservatives [10,12]. The use of essential oils as food preservatives is very promising: (i) they can be considered safe, being subjected to specific protocols for chemical and toxicological analyses, approved guides for their processing and safety restrictions developed by international organisms [14], (ii) the main limitation of their use, represented by their intense aroma that negatively affects the organoleptic properties of foods, is intensively studied to be overcome, with strategies such as active packaging, encapsulation, use in synergy with other antimicrobial agents and/or in combination to other treatment processes [15].

## 2. Results and Discussion

### 2.1. Essential Oils Extractions

Essential oils (EOs) were obtained from different cultivars of *Cannabis sativa* (industrial hemp) by steam distillation. For each cultivar, the extraction at two different times was performed (2 and 4 h). The extraction yield (Table 1) ranged from 0.13 to 1.01% (*w*/*w*). The lower yields observed for cv. Futura 75 at both distillation times are in accordance with those reported in the literature for the same cultivar cultivated in central Italy [6,10].

ANOVA analysis was performed to analyze the different yields of the essential oils, with a multiple comparison test Tukey HSD. The increase in distillation time has resulted in a null or limited increase of the yield, except for the significant increment of Carmagnola Lemon yield and on the contrary for the decrease for Gran Sasso Kush cultivar.

### 2.2. Essential Oils Chemical Composition

The semi-quantitative and qualitative composition of the different EOs analyzed by means of GC–MS is showed in Table 2. The total number of chemical constituents identified in *C. sativa* EOs is 33, of which 12 monoterpenes, 20 sesquiterpenes and 1 cannabinoid.

The qualitative composition of the essential oils of the five hemp cultivars is consistent with the compounds reported by Nissen et al. [12] and Novak et al. [16].

**Figure 1:**
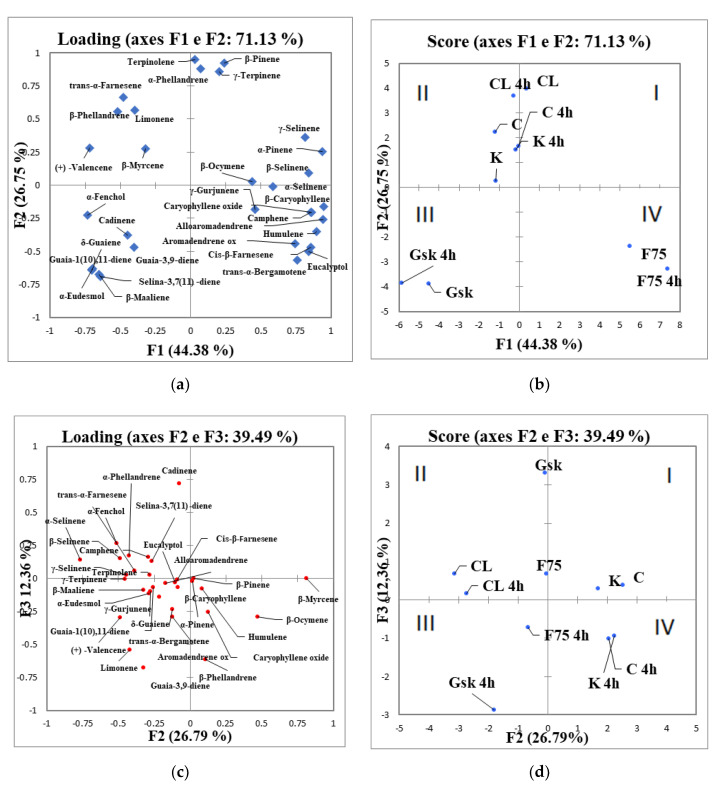
Principal Component Analysis (PCA) of terpenes class obtained after 2 and 4 h of extraction from of the five hemp cultivars. Loading (**a**) and score (**b**) plot of the first two components (explained variance: PC1 = 44.38%; PC2 = 26.75%; total = 71.13%). Loading (**c**) and score plot (**d**) of the second and third components (explained variance: PC2 = 26.75%; PC3 = 12.36%; total = 39.12%). Legend: K, Kompolti at two hours of distillation; K 4 h, Kompolti at four hours of distillation; C, Carmagnola at two hours of distillation; C 4 h, Carmagnola at four hours of distillation; CL, Carmagnola Lemon at two hours of distillation; CL 4 h, Carmagnola Lemon at four hours of distillation; GSK, Gran Sasso Kush at two hours of distillation; GSK 4 h, Gran Sasso Kush at four hours of distillation; F75, Futura 75 at two hours of distillation; F75 4 h, Futura 75 at four hours of distillation.

All data obtained by GC/MS were analyzed by principal component analysis (PCA) to better explain the behavior of terpenes class with five hemp EOs cultivars (Figure 1). Before applying the PCA algorithm, data were expressed as relative abundance (%).

At first, data analysis was performed using PCA of the first two components which concentrate 71.13% of the total variance (PC1: 44.38% and PC2: 26.75%) of the dataset; Figure 1a,b showed a variables (loadings) and samples (scores) plot corresponding to the first two components. In all quadrants of the loading plot, different terpenes contributed to differentiate the analyzed cultivars. Score plot (Figure 1b) showed a different behavior of samples analyzed at two different time of distillation based on the belonging cultivar. Along PC1, a perfect discrimination between Futura 75 and Gran Sasso Kush was achieved. The distillation time did not influence the separation of samples in PCA; in fact, Futura 75 at both times of distillation (2 and 4 h) had the same aroma profile with high concentration of sesquiterpenes such as β-caryophyllene (26–29%), humulene (8–9%) and caryophyllene oxide (2–4%), while α-pinene (16–17%) was representative of the monoterpenes class. Gran Sasso Kush at 2 and 4 h of distillation (Figure 1b, third quadrant), instead, was characterized by minor sesquiterpenes such as guaia-1(10),11-diene (5–7%), β-maaliene (5–7%), α-eudesmol (4–6%), δ-guaiene (3–5%), γ-cadinene (1–11%), guaia-3,9-diene (0–11%) and selina-3,7(11)-diene (14–15%). Along PC2 (Figure 1b), Carmagnola and Kompolti, at 2 and 4 h of distillation, had the same aroma profile with high concentration of β-myrcene (24–41%), while Carmagnola Lemon is separated from the other cultivars, having a high concentration of terpinolene (21–23%).

On the other hand, our aim was to evaluate the aroma profile at different distillation times; for this reason, the PC3 was studied.

Figure 1c,d showed a variables (loadings) and sample (scores) plot corresponding to the second and third components which concentrate 39.12% of the total variance (PC2: 26.75% and PC3: 12.36%) of the dataset. PC3 showed a good discrimination among the samples after 2 and 4 h of distillation (Figure 1d). Along PC3, in the first and second quadrant of the plot (Figure 1d), all the samples analyzed at 2 h of distillation were present, while the samples analyzed after 4 h of distillation were in the third and fourth quadrant (Figure 1d). This separation was due to a variation of content of terpenes (mono and sesquiterpenes) and the increment of cannabidiol (CBD) concentration (Table 2) in all essential oils at 4 h of distillation. Moreover, for some samples, new terpenes components appeared at four hours of distillation such as β-ocymene (2–3%) in Kompolti and Carmagnola, and limonene (9%) and guaia-3,9-diene (11%) in Gran Sasso Kush. Carmagnola Lemon at 4 h presented similar terpenic profile of its oil at 2 h of distillation, except for the increment of CBD (Table 2).

**Figure 2:**
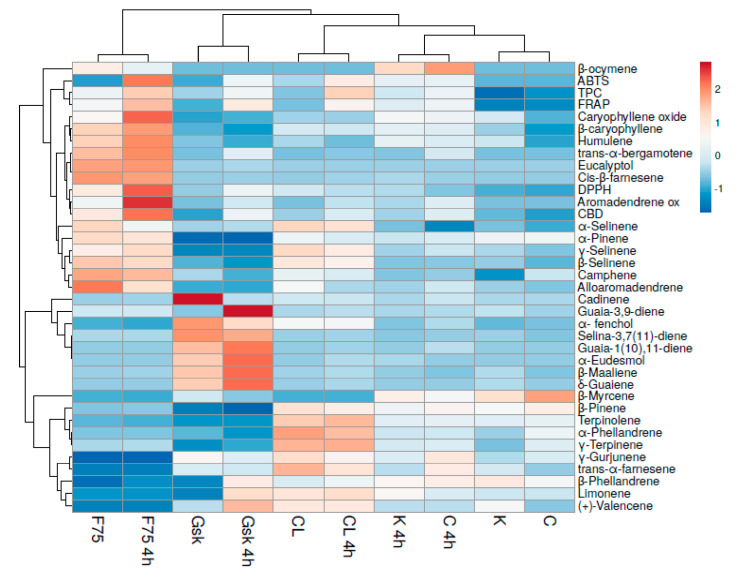
Heatmap diagram based on the relative abundances of the VOCs compounds and the results of the antioxidant activity for each hemp cultivars. Rows in the heatmap represent different volatile compounds and antioxidant activity data, while columns represent different cultivars. The color intensity is proportional to the abundance of VOCs, from blue (the lowest concentration) to red (the highest concentration). Legend: FT: Futura 75; GSK: Gran Sasso Kush; CL: Carmagnola Lemon; C: Carmagnola; K: Kompolti; 4 h: 4 h of distillation; without 4 h: 2 h of distillation.

The correlations among the amounts of the detected volatile fraction compounds of the EOs and the five hemp cultivars were also illustrated through a heatmap diagram (Figure 2).

In details, the heatmap plot graphically depicted the relative abundances of whole VOCs (volatile organic compounds) in the 10 samples at different times of extraction (2 and 4 h), through the gradation from red (high levels) to blue (low levels). The clustering dendrogram of the heatmap diagram grouped the samples reflecting what already highlighted by the PCA.

The same classification is confirmed as the different abundance of VOCs was in relation to the extraction time, in particular for F75 4 h, Gsk 4 h and CL 4 h, where the compounds characterizing the cultivar are in higher quantities than the respective samples at lower time of extraction.

Briefly, the results obtained through PCA and heatmap cluster analysis clustered the various cultivars on the basis of the aromatic profile and the relative abundance of the various VOCs detected. In fact, F75, GSK and CL cultivars had a different qualitative pattern and did not show any significant variation based on the extraction time, obtaining only quantitative differences after two or four hours of extraction. On the contrary, cultivars C and K showed similar aromatic profiles only on the basis of the extraction time, as demonstrated by the fact that the two cultivars at 2 and 4 h cluster together.

The concentration of the mono- and sesqui-terpenic components found in the different cultivars are in agreement with some data reported in the literature [12,17,18]. It has to be considered that Gran Sasso Kush and Carmagnola Lemon varieties are new cultivars, only recently cultivated; for this reason, no data regarding the terpene profile have been described in literature and thus our results are the first report about the chemical composition of these essential oils.

A great variability of the terpenic composition of essential oils was revealed by our data, in accordance with other authors which justified this trend as influenced by various factors, such as the period of collection, genetic characteristics, extraction and processing of the material [6,9]. In our study, the variation of the distillation time caused the stress of the matrix to the point of extracting more or less components and thus to significantly increase the variability of the composition. In literature, no data on a comparative investigation about the influence of different distillation time on the chemical composition are reported.

### 2.3. Antioxidant Capacity (AOC) Evaluation

The results of the total phenolic content (TPC) and antioxidant activity (AOC) FRAP, DPPH and ABTS assays are presented in Table 3. To investigate the different behavior of all the samples, an ANOVA analysis was performed, with a multiple comparison test Tukey HSD (showed in Table 3).

The essential oils after four hours generally showed a high concentration of total polyphenols (*p* < 0.05), except for Kompolti, while, among the samples at two hours, only Futura 75 EO had a high concentration of polyphenols. The antioxidant activity showed the same trend as the TPC, with the exception of the lower DPPH values for Kompolti, Gran Sasso Kush and Carmagnola at two hours.

Therefore, the data showed an interesting antioxidant potential of the hemp essential oils at four hours of distillation with respect to the essential oils at two hours. The different assays established that Futura 75 essential oil at four hours had the maximum antioxidant capacity among the other essential oils, distinguishing itself for the GAE values of 51.71 for the total content of polyphenols, the Trolox values of 11.70 mg for free radical scavenging activity (DPPH), 706.25 mg of Trolox for dosage of antioxidant power of ferric reduction (FRAP) and 137.23 mg of Trolox for radical cation discoloration test (ABTS).

A Pearson correlation test was conducted between the AOC and TPC data and the results of the coefficients obtained were presented in Table 3. The values showed a strong positive correlation of the total phenolic content with antioxidant activity; therefore, the detected antioxidant activity could be mainly attributed to the polyphenols evaluated in the EOs.

Furthermore, the antioxidant potential of EOs at four hours may be associated to the activity of terpenes. In fact, it has been previously shown that the species rich in caryophyllene, humulene and caryophyllene oxide possessed an appreciable antioxidant activity [19,20,21,22]. This would justify the higher TPC and AOC values in both the distillation times of Futura 75 and in the other cultivars (Carmagnola, Kompolti and Caramagnola Lemon and Gran Sasso Kush) after four hours of distillation. Bakkali et al. [23], have showed that the antioxidant properties of EOs cannot be related only to their major constituents. Other minor components, such as β-myrcene, linalool for monoterpenes class or minor sesquiterpenes can also contribute in a synergic way to the obtained antioxidative activity [24,25,26,27]. In fact, Gran Sasso Kush had a rich profile of minor sesquiterpene components already discussed in point 2.2, which contributes to the antioxidant efficiency.

The TPC and antioxidant activity data were included in the heatmap presented in Figure 2. While TPC appears to be strongly correlated to ABTS and FRAP, on the contrary DPPH is correlated to the content of aromadendrene oxide and CBD. This last result has been already highlighted by Borges et al. [28] who affirmed that the antioxidant activity of all essential oils also depends on the concentration of CBD, attributed to its chemical structure [28].

### 2.4. Antimicrobial Activity Evaluation

The antimicrobial activity of the EOs was analyzed by determining their MIC and MBC values on a pool of 10 strains, as shown in Table 4.

The strains were selected among pathogenic (*Salmonella enterica* serovar Enteritidis and *Salmonella enterica* serovar Typhimurium, *L. monocytogenes* and *S. aureus*) and spoiling (*P. fluorescens*, *B. thermosphacta* and *E. faecium*) bacteria commonly isolated from food products of different origins, to have an overview of the antimicrobial potential of the selected essential oils.

The different cultivars and, above all, the different distillation times have significantly influenced the antimicrobial activity of the oils. This variability could probably derive from a synergism between the different bioactive compounds (such as terpenes, cannabinoids) presented in this rich phytocomplex that make them active against microorganisms with different characteristics, such as Gram-positive or Gram-negative bacteria. Iseppi et al. [18] have demonstrated that antimicrobial activity of hemp essential oils can be mainly attributed to sesquiterpenes compounds.

While after two hours of distillation, the EOs were ineffective against *L. monocytogens* and *S. aureus*, after 4 h of distillation, except for Futura 75, they exhibited a stronger antibacterial action against *L. monocytogenes* ATCC 19114 and *S. aureus* ST 47 with MIC values also confirmed by MBC. Previous studies reported the antimicrobial effect only of Kompolti and Carmagnola cultivars on these bacteria, but in some cases with different methods, therefore a comparison is not possible [12,16,18]. However, Iseppi et al. [18] suggested a scarce effect on *S. aureus* and a greater antimicrobial action on *L. monocytogenes.* Futura 75 instead, in which the different distillation times did not involve a variability of response, had no effects against these Gram-positive microorganisms, but presented a good antimicrobial activity against other Gram-positive bacteria, such as *B. thermosphacta* B1 and *E. faecium*, together with all the other essential oils at two hours. The behavior of the Futura 75 is justified by the antimicrobial activity already demonstrated in previous literature. In fact, Nissen et al. [12], and other authors had observed in this cultivar a good activity particularly against *Enterococcus* spp. However, despite this non-homogeneity in the activity against Gram-positive, good results were observed for all the oils, against Gram negative bacteria and in particular on *P. fluorescens* P34. This is a significant result, as in previous research, hemp essential oils of different cultivars were considered as almost ineffective on Gram-negative bacteria [18]. In fact, Gram-negative bacteria generally show a greater resistance because of the composition of the cell wall and are usually difficult to eradicate [29]. The best antimicrobial performance was observed for GSK, particularly after 2 h of distillation, with low MIC values and a wider effect on the tested strains, if compared with the other EOs. The different effect observed after 2 or 4 h of distillation is worth of deeper investigation, nevertheless the chemical composition is certainly involved, as in Figure 1d, the two EOs appears on the opposite sides. In fact, some studies attribute the antimicrobial activity of essential oils to the activity of the terpenes and cannabidiol [30]. Furthermore, in a study regarding numerous chemotypes of *C. sativa*, an increase in antimicrobial activity against some bacteria has been demonstrated thanks to the high relationship between CBD and THC [31] and to the sesquiterpenes [30]. It should be noted that most of our samples at 2 h had a good CBD concentration which increased with the samples at 4 h of distillation. This synergy between the components has led to a more pronounced antibacterial action in some oils, in particular Gran Sasso Kush EOs, as previously described. In fact, this cultivar has a greater cannabinoid and sesquiterpene concentration, in particular of some minor sesquiterpenes components (such as γ-cadinene, selina-3,7(11)-diene) with activity against pathogens, as previously demonstrated by other authors [32]. As regards the bactericidal effect analyzed, it generally remained consistent only for oils at four hours compared to those at two hours, against suggesting an effect of the chemical composition.

The biodiversity of the strains used in these analyses could explain the different behavior shown with respect to the same treatment, which could be strain-specific. Regarding the influence of the essential oils’ composition on bioactivity, not only the major compounds are responsible for antimicrobial activity, as already demonstrated by testing the effect of these compounds alone on several bacteria [18], but often molecules in small amounts could act in synergy, promoting the antimicrobial effect or, on the contrary, acting as antagonists of the biological activity. In fact, the biological activity of essential oils is normally attributed to the most significant molecule(s) based on the composition, but these single purified molecules, if used alone, do not possess the same biological activity, which is attributed instead to the combination of many different molecules collectively affecting the biological activity [33]. These interactions could explain the different activity observed in this study in essential oils of the same cultivars after different distillation times. However, the chemical composition of these essential oils is quite complex and it is almost impossible to determine the precise synergy or antagonism responsible of the greater or the reduced antimicrobial effect observed in the same essential oils at different distillation times.

## 3. Materials and Methods

### 3.1. Plant Material

Dry inflorescences of *Cannabis sativa* cv. ‘Futura 75’ (F75), ‘Carmagnola Lemon’ (CL) and ‘Gran Sasso Kush’ (GSK) were obtained from Hemp Farm Italia, Tortoreto (TE, Italy) while ‘Carmagnola’ (C) and ‘Kompolti’ (K) ones were obtained from MAD BIOFARM SS, Avezzano (AQ, Italy). Plants were open field cultivated in Abruzzo territory (central Italy), starting from certified seeds. Inflorescences were collected during the flowering period (September-October), dried in dark conditions at room temperature (20–25 °C), with controlled relative humidity (45–55%) and stored in the same conditions until processing. Dried inflorescences were also characterized by an accredited laboratory for the contents of main cannabinoids, to assure safe and legal Total THC levels requested by Italian law regulations (2017/1155) [34] (Total THC < 0.2%).

### 3.2. Essential Oils Distillation

In this case, 200–300 g of dried inflorescence were subjected to steam distillation using an essential oils extractor (E0105 12lt Plus, Albrigi Luigi s.r.l.—Verona, Italy). For each hemp matrix, two different essential oils at two different extraction time (2 and 4 h) were obtained. Each distillation was repeated three times. The essential oils were collected and transferred in amber crimp top vials, dried over sodium sulfate, then subjected to an argon conditioning, sealed and stored in darkness at 4 °C.

The distillation yields were calculated according to the Equation (1):

Yield (% *w*/*w*) = (weight of EO (g)/mass dried matrix (g)) × 100 (1)

considering the weight (g) of the EO and the mass (g) of the dried material. The results of the yield were expressed as the average of two replicates of distillation.

### 3.3. Chemical Characterization

GC-MS analysis of essential oils was performed by using a Clarus 580 GC (PerkinElmer—Waltham, MA, USA) equipped with a fused silica Zebron- ZB-SemiVolatile column (30 m × 250 μm × 0.25 μm—Phenomenex, Torrance, CA, USA), coupled to a Clarus SQ 8 S GC/MS (PerkinElmer—Waltham, MA, USA). The oven temperature program was set as follows: starting temperature 50 °C (holding 1 min), ramp 7 °C/min to 145 °C (hold 5 min), ramp 4 °C/min to 175 °C and ramp 7 °C/min to 250 °C (hold 5 min); the carrier gas was Helium at 1 mL/min flow; the injector temperature and the transfer line temperature were set at 250 °C. The essential oil was diluted 1:50 in hexane, and 1 μL of the solution was injected in split ratio 1:50. The analysis was repeated in triplicate for each sample.

The semi-quantitative characterization was carried out through Turbomass 6.1.0.1963 software (PerkinElmer—Waltham, MA, USA). The identification of the main components (α-pinene, β-pinene, limonene, terpinolene, humulene, β-myrcene, β-caryophyllene and caryophyllene oxide) was performed by the comparison of the retention time with those of pure reference material (Sigma-Aldrich, St. Louis, MO, USA). The unknown compounds were identified by matching the obtained spectra with the NIST Mass Spectral Library 2.0 (NIST—Gaithersburg, MD, USA) and confirmed by comparison of the retention index (RI) with those retrieved from http://webbook.nist.gov/chemistry/ (accessed on 4 August 2021). A mix of *n*-alkanes, ranging from octane (C8) to triacontane (C30) was obtained from Supelco (Bellefonte, CA, USA) and injected using the analytical conditions above reported to determine the retention index (RI) as proposed by Lee et al. [35].

A semi quantification analysis was made by peak area normalization without response factors. Relative abundances (%) were the mean of three replicates.

### 3.4. Antioxidant Capacity and Total Phenolic Content

For antioxidant capacity (AOC) and total phenolic content (TPC) assessments, 0.2–0.6 mg/mL of methanolic solutions of the essential oils were subjected to spectrophotometric assays, carried out by means of Lambda Bio 20 UV/Vis spectrophotometer (Perkin Elmer, Waltham, MA, USA).

AOC was evaluated using FRAP [36] (ferric reducing antioxidant power), ABTS (2,2′-azino-bis(3-ethylbenzothiazoline-6-sulphonic acid) [37] and DPPH (2,2-diphenyl-1-picrylhydrazyl) [38] methods. For FRAP, ABTS and DPPH assays, Trolox (6-hydroxy-2,5,7,8-tetramethylchroman-2-carboxylic acid) was employed as reference standard and results were expressed as mg Trolox equivalent (TE)/g essential oil (EO) as mean of three replicates.

TPC was determined by means of Folin-Ciocâlteu method [39]. Results were expressed as mg Gallic acid equivalents (GAE)/g essential oil (EO) as mean of three replicates.

### 3.5. Antimicrobial Activity

To assess the antimicrobial activity of the essential oils, 10 strains belonging to the collection of the Faculty of Bioscience and Technology for Food, Agriculture and Environment, University of Teramo, were employed: three strains of *L. monocytogenes* (ATCC 7644, ATCC 19114 and LM 4), two strains of *S. aureus* (STA 32, STA 47), *P. fluorescens* P34, *B. thermosphacta* B1, *S.* Enteritidis S2, *S.* Typhimurium S4 and *E. faecium* ATCC 19434). Strains growth and standardization conditions, as well as Minimal Inhibitory Concentration (MIC) and Minimum Bactericidal Concentration (MBC) determinations were previously reported [17].

### 3.6. Statistical Analysis

Data obtained, expressed as means ± standard deviations, were subjected to ANOVA (analysis of variance), and a Tukey’s HSD post-hoc test was applied at *p* < 0.05, using Microsoft Xlstat 2016 statistical software (Addinsoft, Paris, France). Correlations between TPC and AOC results were calculated using Microsoft Xlstat 2016 statistical software (Addinsoft) by means of Pearson Correlation. The chemical composition data and the antimicrobial activity results were analyzed by means Principal Component Analysis (PCA) and using Microsoft Xlstat 2016 statistical software (Addinsoft). The heatmap was obtained by Clustvis [40].

## 4. Conclusions

In this study, for the first time, the effect of the distillation time on the biological performance of essential oils was investigated.

Hemp EOs, at two and four hours of distillation, have been obtained from the dry inflorescences of five different cultivar, grown in the Abruzzo region. Among them, Gran Sasso Kush and Carmagnola Lemon are new cultivar, here described for the first time.

The distillation time influenced not uniquely the composition, extracting more or less components thus increasing their variability, but also the biological activity. Anyway, the EOs at four hours of distillation showed a more interesting antioxidant activity with respect to the EOs at two hours, with a maximum observed for Futura 75, demonstrating the synergic effect of the terpenic compounds, including the minor ones, and the cannabinoidic component. The response of antimicrobial activity in ratio of time, instead, was variable. The GSK cultivar showed the best antimicrobial performance, in terms of lower MIC and wider effect in comparison with the other EOs. Moreover, the observed activity of all oils against Gram negative bacteria, in particular for *P. fluorescens* P34, is a very interesting result. The variability of the effect of the distillation time on the antimicrobial activity requires a more in-depth and detailed study, aimed at relating the changes of the chemical components with the antimicrobial effect. Nevertheless, our results suggest that the time of distillation could be modified depending on the subsequent application of the essential oil.

## Figures and Tables

**Table 1:** EOs extraction yields at different distillation times for different cultivars ^1^.

Sample	Variety	Distillation Time (hours)	EO Yield (%)
K	Kompolti	2	0.71 ^c^
K 4 h		4	0.72 ^c^
C	Carmagnola	2	0.58 ^e^
C 4 h		4	0.58 ^e^
CL	Carmagnola Lemon	2	0.61 ^de^
CL 4 h		4	0.85 ^b^
GSK	Gran Sasso Kush	2	1.01 ^a^
GSK 4 h		4	0.64 ^d^
F75	Futura 75	2	0.13 ^f^
F75 4 h		4	0.16 ^g^

^1^ Results followed by the same case-letter are not different according to Tukey’s HSD post-hoc test (*p* > 0.05). Legend: K, Kompolti at two hours of distillation; K 4 h, Kompolti at four hours of distillation; C, Carmagnola at two hours of distillation; C 4 h, Carmagnola at four hours of distillation; CL, Carmagnola Lemon at two hours of distillation; CL 4 h, Carmagnola Lemon at four hours of distillation; GSK, Gran Sasso Kush at two hours of distillation; GSK 4 h, Gran Sasso Kush at four hours of distillation; F75, Futura 75 at two hours of distillation; F75 4 h, Futura 75 at four hours of distillation.

**Table 2:** Gas Chromatography-Mass Spectrometry (GC-MS) characterization of *C. sativa* essential oils ^1^.

RI ^2^	Compounds	K	K 4 h	C	C 4 h	CL	CL 4 h	Gsk	Gsk 4 h	F75	F75 4 h
915	α-pinene	12.31 ± 0.26	9.13 ± 0.94	12.75 ± 0.31	10.38 ± 0.34	12.61 ± 0.27	10.57 ± 1.16	1.022 ± 0.04	0.69 ± 0.04	17.36 ± 0.96	16.48 ± 1.55
932	camphene	0.09 ± 0.12	0.17 ± 0.00	0.23 ± 0.01	0.18 ± 0.01	0.30 ± 0.02	0.26 ± 0.02	0.20 ± 0.01	0.13 ± 0.01	0.51 ± 0.11	0.48 ± 0.03
959	β-pinene	4.64 ± 0.28	4.36 ± 0.04	5.04 ± 0.05	4.84 ± 0.01	5.55 ± 0.16	5.07 ± 0.22	1.532 ± 0.05	1.13 ± 0.05	2.84 ± 0.69	2.88 ± 0.25
969	β-myrcene	32.73 ± 1.69	27.53 ± 5.26	41.42 ± 0.79	23.83 ± 2.76	6.60 ± 0.12	6.52 ± 0.38	16.41 ± 0.35	10.85 ± 1.64	6.33 ± 0.51	5.85 ± 0.41
988	α-phellandrene	0.17 ± 0.22	0.26 ± 0.02	0.39 ± 0.02	0.27 ± 0.00	0.76 ± 0.03	0.69 ± 0.01	0.104 ± 0.00	0.01 ± 0.00	0.14 ± 0.03	0.14 ± 0.01
1011	limonene	4.32 ± 0.02	6.57 ± 0.06	4.19 ± 0.04	5.05 ± 0.10	8.02 ± 0.46	8.62 ± 0.34	0.413 ± 0.00	8.55 ± 0.90	1.21 ± 0.29	1.33 ± 0.11
1015	β-phellandrene	0.18 ± 0.24	0.31 ± 0.01	0.29 ± 0.01	0.33 ± 0.00	0.23 ± 0.03	0.28 ± 0.05	0.049 ± 0.00	0.34 ± 0.02	0.01 ± 0.07	0.06 ± 0.00
1016	eucalyptol	-		-	-	-	-	-	0.03 ± 0.00	0.61 ± 0.14	0.75 ± 0.05
1027	β-ocymene	-	2.21 ± 0.09	-	2.85 ± 0.01	-		-		1.47 ± 0.40	1.12 ± 0.07
1039	γ-terpinene	0.12 ± 0.16	0.25 ± 0.01	0.27 ± 0.00	0.26 ± 0.01	0.59 ± 0.03	0.60 ± 0.01	-	0.05 ± 0.01	0.17 ± 0.04	0.18 ± 0.01
1067	terpinolene	11.04 ± 0.81	10.82 ± 0.07	12.04 ± 0.01	11.55 ± 0.36	21.36 ± 0.32	22.83 ± 3.33	0.176 ± 0.01	0.13 ± 0.00	2.65 ± 0.66	2.31 ± 0.25
1101	α-fenchol	0.14 ± 0.19	0.21 ± 0.28	0.17 ± 0.01	0.37 ± 0.01	0.85 ± 0.01	0.84 ± 0.03	1.662 ± 0.08	1.26 ± 0.03	0.04 ± 0.00	-
1390	β-caryophyllene	16.31 ± 1.25	19.96 ± 1.09	12.84 ± 0.48	19.36 ± 0.31	18.59 ± 0.44	18.06 ± 0.24	14.12 ± 0.08	12.74 ± 0.23	26.27 ± 5.75	29.09 ±1.73
1400	trans-α-bergamotene	-	0.02 ± 0.00	-	0.37 ± 0.02	-	0.06 ± 0.01	-	0.50 ± 0.01	2.61 ± 0.79	1.50 ± 0.89
1416	cis-β-farnesene	-	0.14 ± 0.03	-	0.02 ± 0.01	-	0.10 ± 0.01	-	0.03 ± 0.03	2.67 ± 0.81	3.04 ± 0.17
1423	humulene	5.31 ± 0.16	6.11 ± 0.64	3.76 ± 0.19	5.73 ± 0.13	4.86 ± 0.17	4.32 ± 0.26	4.47 ± 0.02	3.95 ± 0.01	8.01 ± 2.01	9.13 ± 0.78
1428	alloaromadendrene	0.21 ± 0.01	0.22 ± 0.00	0.17 ± 0.01	0.34 ± 0.01	0.56 ± 0.02	0.24 ± 0.33	0.053 ± 0.00	0.04 ± 0.00	1.13 ± 0.32	0.79 ± 0.15
1452	γ-selinene	0.25 ± 0.01	0.24 ± 0.06	0.19 ± 0.01	0.31 ± 0.01	0.71 ± 0.04	0.59 ± 0.04	-	-	0.57 ± 0.05	0.82 ± 0.01
1459	β-selinene	0.66 ± 0.02	0.59 ± 0.18	0.38 ± 0.01	0.62 ± 0.04	2.25 ± 0.10	1.99 ± 0.17	0.345 ± 0.01	-	2.80 ± 0.67	2.59 ± 0.31
1462	(+)-valencene	0.14 ± 0.01	0.08 ± 0.05	0.06 ± 0.00	0.08 ± 0.00	0.17 ± 0.02	0.17 ± 0.02	0.082 ± 0.01	0.22 ± 0.00	-	-
1466	α-selinene	0.41 ± 0.04	0.39 ± 0.08	0.23 ± 0.02	-	1.59 ± 0.06	1.46 ± 0.11	0.544 ± 0.01	0.60 ± 0.02	1.36 ± 0.41	1.05 ± 0.22
1391	γ-gurjunene	0.25 ± 0.01	0.33 ± 0.04	0.32 ± 0.02	0.50 ± 0.03	0.54 ± 0.03	0.44 ± 0.02	0.429 ± 0.01	0.34 ± 0.02		
1408	γ-cadinene	0.94 ± 0.12	0.93 ± 0.14	0.89 ± 0.02	1.49 ± 0.09	1.75 ± 0.07	1.51 ± 0.14	11.22 ± 0.10	1.27 ± 0.06	0.6 ± 0.04	0.70 ± 0.07
1413	guaia-3,9-diene	0.92 ± 0.11	0.45 ± 0.13	0.29 ± 0.01	0.61 ± 0.04	0.57 ± 0.02	0.74 ± 0.07	-	10.56 ± 0.03	1.42 ± 0.20	1.37 ± 0.01
1418	selina-3,7(11)-diene	1.21 ± 0.25	0.55 ± 0.14	0.42 ± 0.03	0.80 ± 0.06	1.07 ± 0.04	1.28 ± 0.15	14.64 ± 0.05	13.36 ± 0.16	1.66 ± 0.41	1.59 ± 0.25
1435	trans-α-farnesene	0.62 ± 0.01	0.51 ± 0.13	0.41 ± 0.02	1.01 ± 0.12	1.38 ± 0.08	1.12 ± 0.22	0.65 ± 0.02	0.61 ± 0.05	-	-
1458	caryophyllene oxide	1.54 ± 0.16	2.05 ± 0.48	0.73 ± 0.03	1.92 ± 0.23	1.15 ± 0.05	1.09 ± 0.20	0.56 ± 0.00	0.71 ± 0.07	2.12 ± 0.57	3.98 ± 0.53
1472	guaia-1(10),11-diene	-	0.02 ± 0.01	-	0.54 ± 0.06	-	0.22 ± 0.05	5.22 ± 0.10	6.71 ± 0.38	-	-
1497	β-maaliene	0.82 ± 0.07	0.39 ± 0.08	0.19 ± 0.01	0.17 ± 0.01	0.58 ± 0.00	0.79 ± 0.16	5.25 ± 0.13	7.11 ± 0.26	0.46 ± 0.02	0.58 ± 0.15
1508	aromadendrene oxide	-	0.13 ± 0.04	-	0.34 ± 0.05	-	0.19 ± 0.04	-	0.27 ± 0.03	0.50 ± 0.01	1.51 ± 0.01
1529	α-eudesmol	-	0.24 ± 0.06	-	0.01 ± 0.01	-	0.36 ± 0.09	4.36 ± 0.10	6.24 ± 0.43	-	-
1539	δ-guaiene	0.29 ± 0.01		-	-	0.02 ± 0.00	0.18 ± 0.05	3.17 ± 0.10	4.51 ± 0.27	-	-
2434	CBD	2.08 ± 0.08	2.92 ± 2.30	1.16 ± 0.10	4.48 ± 2.36	2.84 ± 0.30	4.32 ± 3.63	1.33 ± 0.11	5.23 ± 1.46	6.84 ± 0.01	10.5 ± 0.39
	Total Identified	86 ± 1.20	85 ± 1.11	87 ± 0.52	88 ± 1.14	88 ± 0.58	88 ± 0.77	86 ± 0.86	86 ± 1.70	88 ± 0.48	88 ± 0.95

^1^ Results are expressed as relative abundance ± standard deviation (*n* = 3). ^2^ RI, experimental retention index referred to C8–C40 n-alkane mixture standard. - Not revealed. Legend: K, Kompolti at two hours of distillation; K 4 h, Kompolti at four hours of distillation; C, Carmagnola at two hours of distillation; C 4 h, Carmagnola at four hours of distillation; CL, Carmagnola Lemon at two hours of distillation; CL 4 h, Carmagnola Lemon at four hours of distillation; GSK, Gran Sasso Kush at two hours of distillation; GSK 4 h, Gran Sasso Kush at four hours of distillation; F75, Futura 75 at two hours of distillation; F75 4 h, Futura 75 at four hours of distillation.

**Table 3:** TPC and AOC (antioxidant activity determined by FRAP, DPPH and ABTS assays) of the different essential oils after 2 and 4 h of distillation ^1^.

	TPC	FRAP	DPPH	ABTS
K	26.78 ± 1.13 ^e^	235.33 ± 32.53 ^fg^	8.63 ± 0.28 ^d^	13.76 ± 4.07 ^de^
K 4 h	39.00 ± 0.02 ^bc^	476.67 ± 0.94 ^de^	9.15 ± 0.47 ^bcd^	57.54 ± 4.37 ^c^
C	30.03 ± 0.29 ^de^	250.17 ± 29.93 ^fg^	8.51 ± 0.06 ^d^	13.57 ± 2.45 ^de^
C 4 h	42.82 ± 8.61 ^bc^	519.50 ± 33.47 ^cd^	8.87 ± 0.36 ^d^	59.86 ± 13.25 ^bc^
CL	35.19 ± 1.80 ^cd^	355.83 ± 14.38 ^ef^	9.39 ± 0.32 ^bc^	30.52 ± 3.80 ^d^
CL 4 h	51.26 ± 4.60 ^a^	563.08 ± 1.30 ^bc^	9.18 ± 0.27 ^bcd^	82.53 ± 18.63 ^b^
GSK	36.61 ± 3.52 ^bcd^	320.58 ± 1.538 ^ef^	8.94 ± 0.49 ^cd^	7.67 ± 3.37 ^de^
GSK 4 h	43.51 ± 0.70 ^b^	595.92 ± 17.80 ^b^	9.84 ± 0.24 ^bc^	62.71 ± 4.59 ^bc^
F75	42.7 ± 0.58 ^bc^	536.08 ± 15.07 ^bcd^	10.30 ± 0.30 ^b^	2.57 ± 0.92 ^e^
F75 4 h	51.70 ± 4.34 ^a^	706.25 ± 29.11 ^a^	11.70 ± 1.08 ^a^	137.23 ± 27.55 ^a^
	Pearson Correlation Coefficients			
	ASSAY	FRAP	DPPH	ABTS
	TPC	0.99	0.92	0.97

^1^ Results are expressed as mg Gallic acid equivalents (GAE)/g EO for TPC assay; mg Trolox equivalent (TE)/g EO for FRAP assay; mg Trolox equivalent (TE)/g EO for DPPH assay; mg Trolox equivalent/g EO for ABTS assay. The showed values are the mean of three replicates. For the same assay (same column), results followed by the same case-letter are significantly different according to Tukey’ HSD post hoc test (*p* > 0.05). For Pearson Correlation Coefficients: the positive/negative strength of correlation was considered: low for ±0.1 < r < ±0.3, moderate for ±0.3 < r < ±0.7 and strong for r > ±0.7; for values of r < ±0.1 the variables were considered not correlated. Legend: FT: Futura 75; GSK: Gran Sasso Kush; CL: Carmagnola Lemon; C: Carmagnola; K: Kompolti; 4 h: 4 h of distillation; without 4 h: 2 h of distillation.

**Table 4:** Minimum Inhibitory Concentration (MIC) (µL/mL) and Minimum Bactericidal Concentration (MBC) of hemp essential oils against the different strains ^1^.

	**MIC**									
	**K**	**K4 h**	**C**	**C4 h**	**CL**	**CL4 h**	**GSK**	**GSK4 h**	**F75**	**F754 h**
*L. monocytogenes* ATCC 7644	>20	>20	>20	>20	>20	>20	>20	1.25	>20	>20
*L. monocytogenes* ATCC 19114	>20	10	>20	5	>20	2.5	>20	0.625	>20	>20
*L. monocytogenes* LM4	>20	>20	>20	>20	>20	>20	0.625	0.08	>20	>20
*S. aureus* STA 32	>20	>20	>20	5	>20	>20	5	>20	>20	>20
*S. aureus* ST 47	>20	1.25	>20	2.5	>20	>20	0.156	>20	>20	>20
*P. fluorescens* P34	1.25	0.31	2.5	0.31	1.25	2.5	1.25	2.5	1.25	0.31
*B. thermosphacta* B1	1.25	20	2.5	20	1.25	20	1.25	>20	0.31	0.31
*S.* Enteriditis S2	>20	10	>20	>20	>20	>20	>20	>20	>20	>20
*S.* Typhimurium S4	>20	>20	>20	>20	>20	>20	>20	>20	>20	>20
*E. faecium* ATCC 19434	1.25	>20	2.5	>20	1.25	>20	1.25	>20	0.625	0.625
	**MBC**									
	**K**	**K4 h**	**C**	**C4 h**	**CL**	**CL4 h**	**GSK**	**GSK4 h**	**F75**	**F754 h**
*L. monocytogenes* ATCC 7644	>20	>20	>20	>20	>20	>20	>20	1.25	>20	>20
*L. monocytogenes* ATCC 19114	>20	10	>20	5	>20	2.5	>20	0.625	>20	>20
*L. monocytogenes* LM4	>20	>20	>20	>20	>20	>20	2.5	0.08	>20	>20
*S. aureus* STA 32	>20	>20	>20	5	>20	>20	5	>20	>20	>20
*S. aureus* ST 47	>20	1.25	>20	2.5	>20	>20	0.156	>20	>20	>20
*P. fluorescens* P34	>20	0.31	>20	0.31	>20	2.5	>20	2.5	>20	>20
*B. thermosphacta* B1	>20	>20	>20	>20	>20	>20	>20	>20	10	>20
*S.* Enteriditis S2	>20	10	>20	>20	>20	>20	>20	>20	>20	>20
*S.* Typhimurium S4	>20	>20	>20	>20	>20	>20	>20	>20	>20	>20
*E. faecium* ATCC 19434	>20	>20	>20	>20	>20	>20	>20	>20	>20	>20

^1^ Legend: K, Kompolti at two hours of distillation; K 4 h, Kompolti at four hours of distillation; C, Carmagnola at two hours of distillation; C 4 h, Carmagnola at four hours of distillation; CL, Carmagnola Lemon at two hours of distillation; CL 4 h, Carmagnola Lemon at four hours of distillation; GSK, Gran Sasso Kush at two hours of distillation; GSK 4 h, Gran Sasso Kush at four hours of distillation; F75, Futura 75 at two hours of distillation; F75 4 h, Futura 75 at four hours of distillation.

## Data Availability

Not applicable.

## Sample Availability

Samples of the compounds are available from the authors.
